# The Scandal of Out-Patients

**Published:** 1911-10-21

**Authors:** James Ashenden

**Affiliations:** 22 Albemarle Gardens, New Malden


*To the Editor of* The Hospital.

### THE SCANDAL OF OUT-PATIENTS.

*To the Editor of* The Hospital.

Sir,—Allow me to point out and correct the error which I made in my letter published in your last week's issue with regard to the air space. This should have been from 100 to 500 cubic feet per patient, and not 1,000 to 1,500 as stated, the latter being for hospital wards.—Yours faithfully, James Ashenden.

22 Albemarle Gardens,

New Malden, October 13.

